# Disparities in moderate-to-vigorous physical activity among girls and overweight and obese schoolchildren during school- and out-of-school time

**DOI:** 10.1186/s12966-016-0358-x

**Published:** 2016-03-22

**Authors:** Kristie Hubbard, Christina D. Economos, Peter Bakun, Rebecca Boulos, Kenneth Chui, Megan P. Mueller, Katie Smith, Jennifer Sacheck

**Affiliations:** Tufts University School of Medicine, Public Health and Community Medicine, 145 Harrison Avenue, Boston, MA 02111 USA; ChildObesity180, Friedman School of Nutrition Science and Policy, Tufts University, 150 Harrison Avenue, Boston, MA 02111 USA; University of New England, School of Community and Population Health, 11 Hills Beach Rd, Biddeford, ME 04005 USA; Friedman School of Nutrition Science and Policy, Tufts University, 150 Harrison Avenue, Boston, MA 02111 USA

## Abstract

**Background:**

Increasing physical activity (PA) during the school day and out-of-school time are critical strategies for preventing childhood obesity and improving overall health. The purpose of the present investigation was to examine schoolchildren’s volume and type of PA during school-time and out-of-school, compared to national recommendations and differences by sex and weight status.

**Methods:**

This cross-sectional analysis included 517 3^rd^-5^th^ grade schoolchildren from 13 New England elementary schools (October 2013-January 2014). Demographics were collected by parent questionnaire. Measured height and weight were used to categorize child weight status. Accelerometer data were collected over 7 days. PA was coded as total activity counts and minutes of sedentary, light, and moderate-to-vigorous physical activity (SED, LPA, MVPA) during 1) school, 2) weekday out-of-school, 3) weekend, and 4) total daily time. Multivariable mixed models were used to examine associations between sex and weight status and total counts, SED, LPA, and MVPA, controlling for demographics, wear-time, and clustering within schools.

**Results:**

453 participants (60.5 % girls; mean age 9.1 years; 30.5 % overweight/obese) had valid accelerometer wear time (≥3 days, ≥ 10 h/day). Few children achieved 60 min total daily (15.0 %) or school-time (8.0 %) MVPA recommendations. For all time-of-day categories, girls achieved fewer MVPA minutes than boys (*p* < .0001), and overweight/obese participants achieved fewer MVPA minutes than normal/underweight participants (*p* = 0.05). Minutes of LPA declined by grade-level (*p* < .05) and were lower in girls than boys during school-time only (*p* < .05).

**Conclusion:**

Disparities in MVPA by sex and weight status across school and out-of-school time highlight the need for programs with equitable reach.

## Background

Physical activity (PA) during childhood is associated with numerous physical and mental health benefits, including obesity prevention [1] and improvements in cardiovascular fitness [2], bone mineral density [3], self-worth and social engagement [4–6]. The Physical Activity Guidelines for Americans recommend that school-age children engage in 60 min of moderate-to-vigorous physical activity (MVPA) per day [7]; 30 min of which should be achieved within the school day, according to the Institute of Medicine [8]. Despite the established benefits, nationally-representative objective data indicate that fewer than half of children accumulate 60 min of total daily MVPA [9].

Schools play a key role in obesity prevention and PA promotion and are uniquely positioned to reach millions of children [8]. Ideally, the reach of school PA programs and policies would create an opportunity to provide PA equitably irrespective of age, sex, weight, race/ethnicity, or socioeconomic status. In addition, before and after-school hours (i.e., “out-of-school time”) are also promising yet underutilized times to deliver PA interventions to schoolchildren. After-school programs have recently been targeted for PA policies and standards [10] and some programs have adopted voluntary PA standards for children to achieve 30 min of MVPA while in attendance [11].

Previous studies using objective measures of total PA among schoolchildren suggest that girls are less active than boys [12–14], and PA levels decline with age [15, 16]. The association between weight status and PA is inconsistent. While some studies have demonstrated that overweight and obesity is inversely associated with PA, other studies report no association [17–19]. Several studies have examined children’s activity patterns within the segmented school day and during weekends [12, 15], but few have compared PA levels with national recommendations. Furthermore, the association between child-level anthropometric and socio-demographic characteristics on these more specific patterns and the likelihood of meeting national recommendations is not well-established. It is unknown whether school and out-of-school time environments mitigate these observed subgroup differences in PA participation. An improved understanding of the differences in activity levels during school-time and out-of-school time can facilitate the development of focused intervention efforts and strengthen the vital role of schools and communities to promote and provide PA.

Furthermore, while increasing children’s MVPA is a top public health priority, there is a growing interest to understand the potential health benefits conferred by minutes of light-intensity physical activity (LPA). Time spent in LPA has been associated with cardiometabolic benefits among adolescents [20, 21] and fitness among girls [22], but disparities among subgroups in LPA over the segmented school day and week remain largely unexplored. Therefore, the objectives of the present study were to 1) evaluate differences in patterns of PA across the intensity spectrum on weekdays and weekends, with a particular focus on activity during school time and out-of-school time using hourly, time-stamped data and 2) assess the influence of sex and weight status on PA patterns among schoolchildren and the likelihood of meeting national PA recommendations. The overarching hypothesis was that PA disparities by sex and weight status would be less during school-time than time spent out-of-school, including weekends.

## Methods

### Setting and participants

The analysis used baseline data from a school-based physical activity intervention study that was scheduled to begin the following school-year (2014–15). Eligible schools had received PA mini-grants in the denomination of $1000, agreed to take part in an accelerometer study, and had no current PA promotion grants or special initiatives within the school. In total, 13 of 27 eligible public elementary schools in New England agreed to participate (11 Massachusetts, 1 Maine, 1 Vermont; 48 % participation rate). The 13 public schools represent a mix of urban and rural schools, with average class size of 20 students. Participants were 3rd-5th grade students, recruited in Fall 2013 via classroom visits and assemblies. Recruitment packets were sent home with children approximately one week prior to data collection and included the study information sheet and parent informed consent and child assent forms, in English and Spanish. Students who returned recruitment forms before or on the first data collection day were enrolled in the study. Parents and children provided written informed consent and assent, respectively. Data were collected between late October 2013 and early January 2014. The study protocol was approved by the Tufts University Institutional Review Board.

### Measures

#### Socio-demographic data

Parent and child demographic data were collected from a parent-administered, 9-item, pencil-and-paper survey that was included in the recruitment packet. Participants returned to classroom teachers the demographic survey and consent and assent forms, which were later collected by trained research staff on the day of accelerometer administration. Child race/ethnicity was parent-reported based on National Institutes of Health categories [23] and aggregated into six groups: Asian, Black/African American, Hispanic, Native American, non-Hispanic White/Caucasian, and multiracial. Parent-reported free and reduced-price meal eligibility was used as an indicator of socioeconomic status (SES).

#### Anthropometrics

Assessments of height and weight were conducted in a semi-private setting with participants dressed in light clothing. Height was measured in triplicate without shoes to the nearest 1/8 in. using a portable stadiometer (Model 214, Seca Weighing and Measuring Systems, Hanover, MD). Weight was measured in triplicate to the nearest 0.2 kg using a portable digital electronic scale (PS-6600 ST, Befour Inc., Saukville, WI). Body mass index (BMI) was calculated as body weight in kilograms divided by height in meters squared (kg/m^2^) and converted into a percentile and z-score using the Centers for Disease Control and Prevention (CDC) age- and sex-specific growth charts [24]. BMI percentiles were classified accordingly as: <5^th^ percentile as underweight, 5^th^-85^th^ percentile as normal weight, 85^th^-95^th^ percentile as overweight, and ≥95^th^ percentile as obese.

#### Weather conditions

Weather data were collected from the National Oceanic and Atmospheric Administration [25]. The high temperature (continuous) and precipitation (binary: yes/no) were recorded for each day the accelerometers were worn by participants from the weather station nearest to each school.

### Measurement of physical activity

#### Instrumentation

Physical activity was measured by Actigraph GT3X and GT3X+ accelerometers (ActiGraph, LLC, Pensacola, FL), validated and calibrated for use among children [26].

#### Protocol

Participants were outfitted with an accelerometer by trained research staff at scheduled school study visits. Trained research staff showed participants how to properly wear the accelerometer, and printed instructions were sent home. Monitors were attached to adjustable elastic belts and worn over the right hip, consistent with previous studies [9, 12]. Participants were instructed to wear the accelerometer for seven consecutive days during all waking hours, except when bathing or swimming. Participants were also given a 7-day PA log to record the times the monitor was worn and to provide supplemental information about activities that would not be captured by the accelerometers, such as swimming or cycling. Monitors and PA logs were returned after 7 days and collected by research staff.

#### Data reduction

Accelerometers were initialized to sample and store activity counts beginning at 00:00:01 on the first day the participant was instructed to start wearing the device. Stored activity counts from each monitor were downloaded for data reduction and analysis. Accelerometer data were processed as counts per 30-s epoch to more accurately record short, intermittent bursts of activity common among children as compared to longer epoch lengths [27]. Non-wear time was defined as 60 min of zero activity counts (i.e., 120 consecutive zeroes), allowing for 1 min of light activity (two consecutive epochs with 1–99 counts) every hour. Wear time was estimated by subtracting non-wear time from the total daily monitoring time. A day was considered a “valid day” if daily wear-time was ≥10 h. Participants with < three valid wear days were excluded from the analysis.

Epochs were classified into the following PA intensity categories using the cut points developed specifically for children by Evenson and colleagues: sedentary (≤50 counts), light (51–1148 counts), moderate (1149–2005 counts), and vigorous (≥2006 counts) [28]. Hour, time of day, and weekday or weekend were inserted on the accelerometer output. For each valid day, minutes in each intensity category were averaged for each participant across four segments: total daily time (sum of minutes across all valid days divided by number of valid days), school time, weekday out-of-school time, and weekend time. School-time hours were calculated for each participant, based on the specific start and end times of the school day for each day the accelerometer was worn. This specificity allowed for adjustment of school-time hours on days in which students were released early from school or had ½ days. Before-school time was calculated as the time elapsed between 12 AM and the start of the school day. After school-time was calculated as the time elapsed between the end of the school day and 11:59 PM. Weekday out-of-school time was calculated as the sum of before school-time and after-school time. Total wear time (continuous, around the clock) was used on weekends.

### Statistical analysis

Participant demographic characteristics were summarized using descriptive statistics. Differences between participants who completed the accelerometer protocol (*n* = 453) and those with less than 3 valid wear days (*n* = 42) (excluding cases attributable to accelerometer failure in the field or at the time of download) were evaluated using *chi*-square tests for categorical variables (e.g. sex, race/ethnicity, weight status) and *t*-tests for continuous variables. For the multivariable analyses, participants were classified as overweight/obese if their BMI was ≥ the sex- and age-specific 85^th^ percentile. Otherwise, participants were classified as normal/underweight.

Outcomes assessed included total activity counts and minutes spent in each activity intensity category (sedentary [SED], light [LPA], and moderate-to-vigorous [MVPA]). Mixed linear regression models were used to assess differences in total activity counts, SED, LPA, and MVPA during school time and weekday out-of-school time by sex and weight status. SAS PROC MIXED was used to construct random intercept models to account for the clustering of participants within schools. Degrees of freedom were calculated using the between-within method. Race/ethnicity was reduced from six categories to two categories (non-Hispanic White vs. not non-Hispanic White) for the analyses. All models were adjusted for grade; race/ethnicity; SES; average high temperature over the wearing period and number of days of precipitation over the wearing period (both variables specific to each subject’s valid wear days); and average wear-time (e.g., “total daily” models were adjusted for total wear-time; “school time” models were adjusted for school-day wear time). The following interaction terms were tested for all PA outcomes: sex by overweight/obese status, grade by overweight/obese status, grade by sex, and sex by grade by overweight/obese status. General linear models with a logit link were used to calculate the odds of meeting PA guidelines according to overweight/obese status, accounting for clustering of participants within schools and adjusting for previously mentioned covariates. All statistical analyses were conducted using Statistical Analysis Software (SAS), version 9.2 (2009, SAS Institute, Cary, NC). *P*-values less than 0.05 were considered statistically significant.

Two series of sensitivity analyses were conducted. First, outcomes of interest were compared based on a study sample providing 4 or more valid wear days compared to 3 or more valid days. No significant differences in outcomes were found between the two samples, providing justification for using 3 or more valid days as the inclusion criteria for all analyses. Second, we examined potential differences in outcomes when a) establishing three weight categories (i.e., normal/underweight, overweight, obese) vs. two weight categories (i.e., normal/underweight vs. overweight/obese) and b) including vs. excluding underweight children (*n* = 15) from the normal/underweight category. No differences in outcomes were found when including vs. excluding underweight children from the normal/underweight category nor when separating obese children (*n* = 73) out from the overweight/obese category.

## Results

### Study participants

A total of 517 3^rd^-5^th^ grade participants from 13 schools were enrolled in the study and outfitted with an accelerometer. Among these, 17 accelerometers failed in the field, 2 accelerometer files failed to download, 42 participants provided less than 3 valid wear days, and 3 were excluded due to missing anthropometric data, for a final sample size of 453 participants. The mean (standard deviation) age of the study participants for the analyses was 9.1 years (0.9 years), 60.5 were female and 30.5 % were overweight or obese (Table 1). Mean valid wear days (standard deviation) among the study participants with 3 or more valid days (*n* = 453) was 5.8 days (1.4 days) with a mean total daily wear time (standard deviation) of 14.2 h (2.1 h) per day. Average wear times on weekdays and weekends were as follows: school time 6.25 h; weekday out-of-school 7.76 h, and weekends 14.3 h.

**Table 1:** Descriptive Statistics for Accelerometry Sample (*n* = 453)

Age, years (st dev)	9.1 (0.9)	
	N	%
Sex		
Female	274	60.5
Grade		
3^rd^	182	40.1
4^th^	159	35.3
5^th^	112	24.7
Race/ethnicity^a^		
Asian	11	2.4
Black/African-American	22	4.9
Hispanic/Latino	48	10.6
Native American	4	0.9
Non-Hispanic White	337	74.4
Multiracial	15	3.3
Weight Status		
Underweight	15	3.3
Normal weight	300	66.2
Overweight	65	14.4
Obese	73	16.1
Free- & Reduced-Price Lunch Eligible^a^		
Yes	140	30.9
Parent/Guardian Highest Level of Education^a^		
High school graduate/GED or less	94	20.8
Some college or college degree	347	76.6

^a^Percentages do not add up to 100 due to missing data

### PA outcomes

**Fig. 1:**
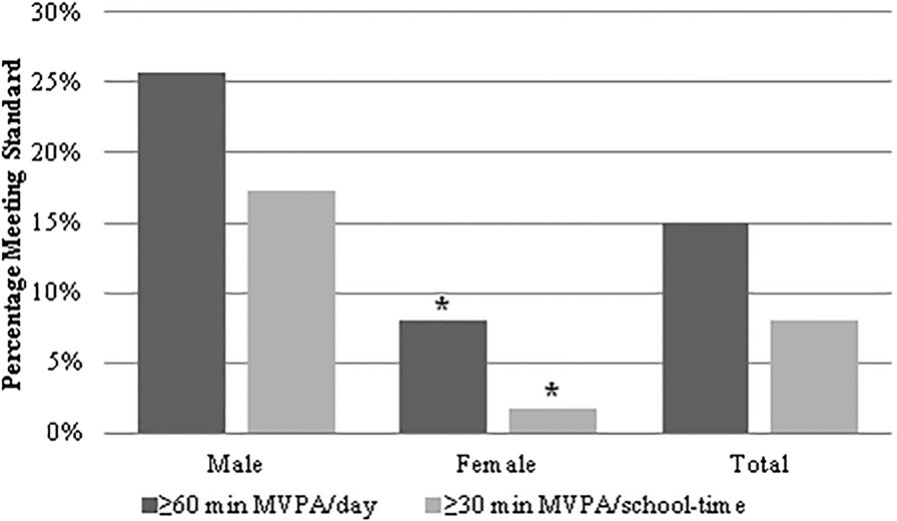
Participants meeting recommendations for 60 min of total daily- and 30 min of school-time MVPA. Based on general linear models adjusted for grade, race/ethnicity, SES, wear-time, weather conditions, and clustering within schools. *p < 0.0001 compared to males

Mean total activity counts and minutes of MVPA, LPA, and SED for the accelerometer sample are displayed in Table 2. Participants accumulated a mean (standard deviation) 42.7 (20.8) minutes of total daily-; 18.1 (8.1) min of school-time-; 24.8 (14.0) minutes of weekday out-of-school-; and 44.7 (34.5) minutes of weekend MVPA. Few participants (*n* = 68, 15.0 %) met recommendations for 60 min of total daily- or 30 min of school-time MVPA (*n* = 36, 8.0 %). Overweight/obese participants were no less likely than normal/underweight participants to meet recommendations for total daily- (11.6 vs. 16.5; *p* = 0.31) and school-time MVPA (8.0 % vs. 8.0 %; *p* = 0.22). However, girls were less likely than boys to meet both the 60 min total daily- (8.0 vs. 25.7; *p* < 0.0001) and the 30 min school-time (1.8 % vs. 17.3 %; *p* < 0.0001) recommendation (Fig. 1). The odds of meeting total daily- or school-time MVPA recommendations did not differ by grade, race/ethnicity, or SES.

**Table 2:** Average total daily activity counts and minutes of MVPA, LPA and SED^a^

	Total daily	School-time	Weekday Out-of-School	Weekend
Total Activity (counts)	380,120 (127,716)	161,513 (50,730)	213,718 (155,472)	400,230 (210,337)
MVPA (minutes)	42.7 (20.8)	18.1 (8.1)	24.8 (14.0)	44.7 (34.5)
LPA (minutes)	312.2 (53.9)	143.8 (32.6)	168.5 (35.0)	311.6 (67.4)
SED (minutes)	486.6 (120.5)	213.9 (37.3)	272.9 (108.6)	499.2 (166.8)

^a^Unadjusted means and standard deviations. Counts and minutes in each intensity category were averaged for each participant across four time-of-day categories: total daily, school-time, weekday out-of-school, and weekends

Associations between volumes and type of PA stratified by sex and weight status are presented in Table 3. We found no significant interactions between sex and weight status, sex and grade, or grade and weight status for all PA outcomes (all *p-*values for interaction terms >0.05). Overweight/obese participants were less active overall than normal/underweight participants; they accumulated significantly fewer total daily activity counts (average weekday and weekends), school-time activity counts, out-of-school activity counts, and weekend activity counts as compared to normal/underweight participants (Table 3). Overweight/obese children achieved significantly fewer minutes of MVPA for the total day (36.6 min vs. 44.5 min, *p* = 0.0002), school-time (16.6 min vs. 19.6 min, *p* = 0.0003), weekday out-of-school (20.9 min vs. 25.5 min, *p* = 0.002), and on weekends (35.6 min vs. 46.5 min, *p* = 0.007) compared to normal/underweight children. There were no differences in SED or LPA minutes for overweight/obese children as compared to normal/underweight children for total daily, school-time, weekday out-of-school, or weekends.

**Table 3:** Associations between MVPA, LPA, and Total Activity Counts and Overweight/Obesity Status and Sex

	Overweight/Obesity status			Sex		
	β coefficient^a^	95% CI	*p*-value	β coefficient^b^	95 % CI	*p*-value
MVPA minutes						
Total daily	−7.8	−11.9–3.7	0.0002	−17.8	−13.8–21.8	<.0001
School-time	−3.0	−4.5–1.4	0.0002	−7.8	−6.3–9.3	<.0001
Weekday Out-of-School	−4.6	−7.5–1.7	0.001	−8.2	−5.4–10.9	<.0001
Weekend^c^	−11.0	−19.0–3.0	0.007	−22.6	−14.8–30.5	<.0001
LPA minutes						
Total daily	2.2	−7.8–12.3	0.66	−9.3	0.5–19.1	0.06
School-time	−0.6	−6.39–5.31	0.85	−9.9	−4.3–15.7	0.0007
Weekday Out-of-School	2.8	−3.6–9.2	0.39	−4.1	2.1–10.4	0.19
Weekend^c^	1.0	−14.3–16.3	0.89	5.7	−8.9–20.3	0.44
Total Activity Counts						
Total daily	−37,395	−63,046–11,743	0.004	−89,813	−64,888–114,819	<.0001
School-time	−14,704	−24,638–4,769	0.004	−40,649	−30.923–50,375	<.0001
Weekday Out-of-School	−21,736	−38,940–4,531	0.02	−42,635	−25,825–59,444	<.0001
Weekend^c^	−52,490	−101,963–3,015	0.03	−109,899	−61,159–158,638	<.0001

^a^Represents difference in PA between overweight/obese participants and normal/underweight participants. Results are based on mixed linear regression models adjusted for grade, sex, race/ethnicity, SES, weather conditions, wear-time, and clustering within schools

^b^Represents difference in PA between girls and boys. Results are based on mixed linear regression models adjusted for grade, weight status, race/ethnicity, SES, weather conditions, wear-time, and clustering within schools

^c^ *n* = 368

Girls were less active overall than boys. Girls achieved significantly fewer total daily activity counts, school-time activity counts, weekday out-of-school activity counts, and weekend activity counts as compared to boys (Table 3). Girls also accumulated fewer minutes of MVPA for the total day (31.7 vs. 49.5 min; *p* < 0.0001), school-time (14.2 vs. 22.0 min; *p* < 0.0001), weekday out-of-school (19.1 vs. 27.3 min; *p* < 0.0001), and weekends (29.7 vs. 52.4 min; *p* < 0.0001). Total daily and school-time LPA minutes were also fewer among girls as compared to boys; no differences in LPA minutes by sex were observed for weekdays, out-of-school or weekends. Girls accumulated more SED minutes during school-time (225.9 vs. 208.13; *p* < 0.0001) and weekday out-of school (280.2 vs. 267.7; *p* < 0.0001) compared to boys, but these differences were not observed for total daily and weekends.

**Fig. 2:**
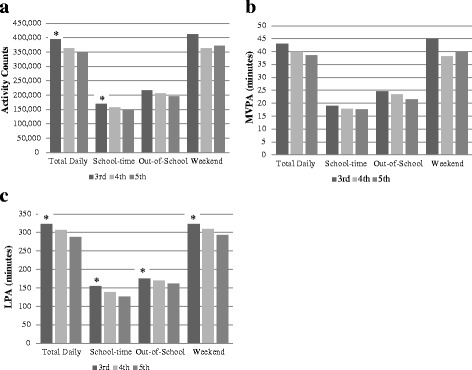
Differences in (**a**) Total Activity Counts, (**b**) MVPA, and (**c**) LPA by Grade Level. Results are based on mixed linear regression models adjusted for sex, weight status, race/ethnicity, SES, weather conditions, wear-time, and clustering within schools. *p < 0.05 compared to 4^th^ and 5^th^ grade

We observed a significant decline in total activity counts with increasing grade level for total daily (*p* = 0.02) and school-time (*p* < 0.0001), but not weekday out-of-school or weekends (Fig. 2a). Minutes of LPA significantly declined with increasing grade level for total daily (*p* < 0.0001), school-time (*p* < 0.0001), weekday out-of-school (*p* = 0.006), and weekends (*p* = 0.009) (Fig. 2c). No significant differences in minutes of MVPA were observed by grade-level (Fig. 2b). However, sedentary minutes significantly increased with increasing grade level for total daily (*p* < 0.0001), school-time (*p* < 0.0001), weekday out-of-school (*p* = 0.006), and weekends (*p* = 0.008). Race/ethnicity and SES was not significantly associated with total activity counts, MVPA, SED, or LPA within the segmented school-day or on weekends. In unadjusted total daily and school-time models, children accumulated fewer minutes of LPA and MVPA on days with precipitation. The effect was attenuated in the fully-adjusted mixed models; precipitation was a significant predictor in the school-time MVPA (*p* = 0.02) and LPA (*p* = 0.03) models only. No effect of temperature was observed.

## Discussion

Contrary to our expectations, children achieved fewer minutes of MVPA during school-time compared to weekday out-of-school hours even though the recommendation is that children achieve at least half of the daily recommendation during school hours. While a previous investigation based on nationally-representative data found that school-time MVPA accounts for the largest proportion of total daily MVPA on weekdays [12], others have found that total activity counts and minutes of MVPA are higher during out-of-school time compared to school-time and lowest during weekends [15, 29]. Furthermore, we had hypothesized that PA disparities by sex and weight status would be minimized by the structured school-day environment, where in theory, the reach of PA programs and policies should be equitable, compared to weekends and out-of-school time. We found MVPA disparities by sex for all measured segments, including school-time, with the greatest disparity in MVPA between boys and girls occurring during weekends. Others have also reported that girls achieve greater minutes of MVPA during out-of-school time (vs. school time) [30], and greater disparities in total activity counts between girls and boys have been ascribed to school-time (vs. out-of-school time and weekends) [15].

Although current PA guidelines specify recommendations that focus on MVPA, the majority of a child’s day is spent in sedentary and light physical activities, with LPA contributing the greatest amount of movement to total activity levels. Contrary to our findings on MVPA, compared with boys, girls achieved similar amounts of LPA on weekends and during out of school hours, but remained significantly lower during school-time. Interestingly, overweight/obese children engage in similar levels of LPA on weekdays during school, out-of-school and on weekends compared to normal weight children, further highlighting that the school-time disparity in LPA exists only between girls and boys.

In addition, minutes of LPA declined with increasing grade level for all segments of the school-day and on weekends, however there was no significant decline in MVPA. It is noteworthy that total activity only declined across grades during school-time hours, but did not significantly decline during weekdays, out-of-school, or on the weekends. While MVPA is currently not ideal, emphasis on total movement should continue to be encouraged as children move through the elementary school years. Targeting opportunities to increase LPA during school-time is highly feasible and is gaining considerable public health interest [21]. Although MVPA recommendations remain of upmost importance, increasing minutes of LPA may be a critical first step for children who are far from meeting PA guidelines, even though the potential health benefits of LPA among children are still emerging [20, 21].

Physical inactivity is a key determinant of childhood obesity and health disparities throughout the life-course [7]. The majority of schoolchildren in this sample from three New England states are not meeting the physical activity guidelines for total daily- and school-time MVPA. Across the entire sample, only 15 and 8 % met these PA guidelines, respectively. It is particularly concerning that in girls, only 8 and 2 % met total daily- and school-time respectively. Our findings and the dearth of research in this area highlight the need to identify and implement school-based, out-of-school time, and weekend PA programs with equitable reach to girls and overweight/obese children. Time spent in PE and recess has been reduced or eliminated in many schools [31], and only six states mandate the Institute of Medicine’s recommended 150 min of PE per week in elementary schools [32]. Standardized testing requirements and budget cuts may contribute to the declines in school-based PA opportunities [31]. In light of these challenges, The Institute of Medicine has recommended a “whole-of-school” approach in which recess [33], in-class PA breaks [34], and integration of PA with the classroom curriculum are highlighted as action areas to increase time spent in MVPA [8].

The present study had several limitations. First, the generalizability of the results should be considered. The sample comprised New England schools and participants who had received PA mini-grants for the following school year and control schools matched on geographic location and socio-demographic variables. Presumably, the schools and participants in our sample who volunteered for the accelerometer study would be more motivated than other schools to provide students with PA during the school day. Therefore, the findings that few of the participants met total daily- or school-time MVPA recommendations are noteworthy. The sample also lacked significant demographic diversity, which precluded the ability to fully investigate the association between race/ethnicity and SES on PA outcomes of interest. Due to sample size constraints, race/ethnicity was reduced to two categories for the analyses. Although previous studies have found PA disparities by race/ethnicity [18], we observed no differences in total activity counts, minutes of MVPA, SED, or LPA by race/ethnicity or SES in the present study. Accelerometry studies based on nationally-representative samples, such as NHANES, indicate a complex relationship between PA and gender, weight status, and race/ethnicity [35]. Future studies examining disparities in volume and type of PA within the school day and weekends should include larger and more diverse samples of schoolchildren to allow full evaluation of these relationships.

Strengths of the present investigation include an evaluation of both volume and type of PA to better understand when and which populations might most benefit from targeted interventions. The study sample and design facilitated the collection of school data specific to each valid wear day for each participant, including start and end times of the school day and weather information. These were important factors to account for in the analyses given the goal to describe differences in PA patterns during school time and out-of-school time and would not be possible to include if using a nationally representative dataset such as NHANES which does not collect this information.

## Conclusion

In summary, fewer than one in seven and one in ten elementary schoolchildren accumulated recommended minutes of total daily- and school-time MVPA, respectively. Girls and overweight/obese children engage in less total movement than boys and normal/underweight children both in school and during out-of-school time. This disparity exists specifically in MVPA, however, in girls there is also a distinct disparity in LPA that exists during the school day that is not evident during out of school-time hours. These data demonstrate that disparities in MVPA transcend school and out-of-school time in both girls and overweight and obese children, which is further exacerbated by lower LPA in girls during school hours. These findings underscore that PA disparities exist both in school and out-of-school and highlight the need to identify and implement school-based PA programs with equitable reach along with emphasizing opportunities for additional MVPA for all children.
